# The Question Respecting the Effects of Climate in Changing the Human Form and Colour; Argued from Facts Collected at Barbadoes

**Published:** 1808-09

**Authors:** 


*The Question respecting the Effects of Climate in changing the Human Form and Colour; argued from Facts collected at Barbadoes; by Dr.* PINCKARD.

IN the part of the island near the tar pits, we happened to call in at a small hut, or cabin, where we met with a large family of Barbadian cottagers; and, with all the inquisitiveness of strangers, we addressed the good people in a multitude of interrogatories, and were highly gratified with their replies. They were living amidst the mountains, apparently shut from the world, and but seldom exposed to the intrusion of strangers. The old dame of the house was nearly seventy years of age. We found her occupied in playful attentions with two of her grand children—two, of seven, of the offspring of her daughter. Making inquiries respecting the old woman's history, we learned that she could trace back her family in regular lineal descent, as far as her great grandfather, the successors of whom have never removed from Barbadoes; so that the children we here saw, were to a certainty as distant as the sixth generation, and probably much more remote, in direct descent, from parents who had always lived in the torrid zone. One of the children was about six—the other eight years old. In fairness of skin, in feature, and in figure, they might have been mistaken for children born in England, or any other temperate climate.

Near Hilloughby hill we met with another cottage family, regularly descended from British parents, of long standing in the island, and having all the features and general appearance of Europeans. The father of this family was sixty years old, and some of his predecessors had lived to upwards of ninety years. We could not trace the pedigree so accurately as in the other family—but this probably was not less ancient, the old man having no knowledge but of his Barbadian predecessors, and not knowing when they first came to the island. The occupation of this family was that of planting a small spot of land with ginger, and raising stock to sell at Bridge-town market. They were poor, like the others, and compelled to labour much in full exposure to the sun. Like the negroes, too, their diet consisted chiefly of vegetables.

At the fort, commanding the entrance of Carlisle bay, are living a man and his wife, both natives of Barbadoes, whose ancestors for generations, beyond all that tradition has traced to them, have resided constantly in the island: sitting sitting round the mother we saw five fine children—their offspring, of face and form as fair as the fairest Europeans.

These facts stand in direct opposition to the speculative doctrines of those who derive the various colours of the human race from climate or locality of residence, together with the concomitant circumstances of diet, and mode of life. Consistent with such opinion it follows, that the offspring of Europeans, living constantly in the torrid zone, and, more particularly, if using the same diet, and exposed to similar habits and occupations, must degenerate, and, in future ages, become negroes.

The three families above-mentioned are, undoubtedly, of the fifth or sixth, or perhaps, a still more distant generation, in direct lineal descent, from parents, originally, English; but whose offspring, through every race, to the present children, have always resided between the tropics. They have moreover, lived in circumstances of mediocrity, exposed to labour, and to the full influence of climate; or have known only the abode of poverty, and by needy fortune have been compelled to use a diet very similar to that of the Africans. Yet is there not an individual among them, who, either in form, feature, or colour, has made even the slightest approach to that change, which a constant residence, through so many generations, must have effected, were their descendants, of future ages, to become of negroe form and hue.

Allowing this change of our species to be as slow and gradual as the warmest advocate of the doctrine might suppose, it were impossible for the mind to conceive a period, when the offspring of Europeans would be broiled into perfect negroes, if no sort of commencement—no mark whatever of deviation—nor any approach to the conversion, could be traced, either in the features, or the skin, of those of the fifth, or sixth, or perhaps of the eighth or ninth generation; after a residence, too, in the successive races, of nearly two hundred years under a tropical sun, and being exposed to most of the other causes, said to promote the expected revolution of their frames!

Children born in England have not fairer skins, nor features more correctly European. The younger have all the cherub face and form of the lovely smiling babes of a temperate climate. Those more advanced are thinner, and bear about them more of that languor, which universally results from long residence in great and constant heat; but still have they no kind of approach to the thickened lip—

the the large mouth—the projecting countenance—the flattened nose—the lengthened head—the wooly hair—or the dark skin of the negroes.

The opinions of the gentlemen of the island seem to be all against the idea of such a conversion of the human body; and we are assured that multitudes of families, in addition to those we have seen, now live in Barbadoes, who in progressive descent, through successive generations, for nearly two hundred years, have resided in the island, without the slightest change being perceptible in their offspring of the present day.

To whatever age the parents may have lived, it is remarkable that, although the face and hands shall have become brown, from immediate exposure to the sun, the other parts of their bodies remain white and unchanged; and not the softest shade—not the slightest tinge of the acquired darkness of hands or face is communicated to the descendants—the children being, invariably, born as perfect whites as those of Europe. If, therefore, it could, for a moment, be admitted that merely the tanned countenance were an approach to the negro state, this being completely extinguished in each succeeding race, it could never advance beyond the feeble change effected in a single generation.

But the very strong and incontrovertible fact with respect to the American Indians, militates so decidedly against this doctrine of conversion, that scarcely another argument can be necessary to its refutation. Although living for unknown ages under the same parallel of latitude as the Africans, and exposed to precisely similar habits and occupations, not an individual of them has ever been known to turn negro, either in skin or feature. Nor, indeed, would it be less reasonable to expect that the negroes of Africa, or those of the West India islands, should change to Indians, than that Indians, or Europeans, should be converted into Africans!

